# Carnosine Did Not Affect Vascular and Metabolic Outcomes in Patients with Prediabetes and Type 2 Diabetes: A 14-Week Randomized Controlled Trial

**DOI:** 10.3390/nu15224835

**Published:** 2023-11-19

**Authors:** Saeede Saadati, James Cameron, Kirthi Menon, Alexander Hodge, Zhong X. Lu, Maximilian de Courten, Jack Feehan, Barbora de Courten

**Affiliations:** 1 Department of Medicine, School of Clinical Sciences, Faculty of Medicine, Nursing and Health Sciences, Monash University, Clayton, VIC 3168, Australia; saeede.saadati@monash.edu (S.S.); kirthimenon7@gmail.com (K.M.); alex.hodge@monash.edu (A.H.); zhong.lu@monashhealth.org (Z.X.L.); 2 Monash Cardiovascular Research Centre, Monash Heart, Monash Health, Clayton, VIC 3168, Australia; 3 School of Health and Biomedical Sciences, RMIT University, Bundoora, VIC 3083, Australia; 4 Monash Health Pathology, Clayton, VIC 3168, Australia; 5 Mitchell Institute for Health and Education Policy, Victoria University, Melbourne, VIC 3011, Australia; maximilian.decourten@vu.edu.au; 6 Institute for Health and Sport, Victoria University, Melbourne, VIC 3011, Australia

**Keywords:** Carnosine, randomized trial, RCT, cardiovascular risk, diabetes, insulin resistance, metabolic health

## Abstract

Atherosclerotic cardiovascular disease (ASCVD) is the leading cause of morbidity and mortality in patients with prediabetes and type 2 diabetes mellitus (T2DM). Carnosine has been suggested as a potential approach to reduce ASCVD risk factors. However, there is a paucity of human data. Hence, we performed a 14-week double-blind randomized placebo-controlled trial to determine whether carnosine compared with placebo improves vascular and metabolic outcomes in individuals with prediabetes and T2DM. In total, 49 patients with prediabetes and T2DM with good glycemic control were randomly assigned either to receive 2 g/day carnosine or matching placebo. We evaluated endothelial dysfunction, arterial stiffness, lipid parameters, blood pressure, heart rate, hepatic and renal outcomes before and after the intervention. Carnosine supplementation had no effect on heart rate, peripheral and central blood pressure, endothelial function (logarithm of reactive hyperemia (LnRHI)), arterial stiffness (carotid femoral pulse wave velocity (CF PWV)), lipid parameters, liver fibroscan indicators, liver transient elastography, liver function tests, and renal outcomes compared to placebo. In conclusion, carnosine supplementation did not improve cardiovascular and cardiometabolic risk factors in adults with prediabetes and T2DM with good glycemic control. Therefore, it is improbable that carnosine supplementation would be a viable approach to mitigating the ASCVD risk in these populations. The trial was registered at clinicaltrials.gov (NCT02917928).

## 1. Introduction

Type 2 diabetes mellitus (T2DM) and its related complications are a leading cause of morbidity and mortality. Diabetes affects 537 million adults globally, estimated to rise to 783 million in the next 22 years [1]. T2DM significantly increases chronic disease risk and is a major risk factor for atherosclerotic cardiovascular disease (ASCVD) [2]. Patients with diabetes have approximately double the ASCVD risk compared to those without diabetes [3]. Importantly, T2DM leads to both microvascular (nephropathy, retinopathy, and neuropathy) and macrovascular atherosclerotic diseases (peripheral vascular disease, ischemic heart disease, coronary artery disease, and stroke) [4].

Endothelial dysfunction is important in the development of atherosclerosis and is commonly seen in prediabetes [5,6]. Endothelial dysfunction can occur independently of diabetes risk factors, including insulin resistance, inflammation, and obesity-related dyslipidemia but diabetes further worsens endothelial dysfunction [7,8]. However, conversely, endothelial dysfunction is the outcome of many diabetic physiologies, including impaired insulin signaling and resistance, hyperglycemia, increased free fatty acids, and decreased bioavailability of nitric oxide (NO), which leads to increased vascular oxidative stress due to decreased vasodilation [7,9]. In addition to microvascular disease manifesting organs, the liver also exhibits low-resistance arterial hemodynamics due to receiving one-third of its blood flow by the hepatic artery [10]. Despite the management of diabetic complications, gaps in therapeutic approaches still exist.

Carnosine (β-alanyl-L-histidine) is an emerging therapeutic agent that can potentially contribute to the management of T2DM and its vascular complications [11,12,13,14]. Several experimental studies revealed that carnosine has benefits in atherogenesis by elimination of lipid peroxidation-derived aldehydes from atherosclerotic lesions and prevention of low-density lipoprotein cholesterol (LDL-C) oxidation [15,16,17]. In addition, carnosine can ameliorate dyslipidemia by down-regulating sterol regulatory element-binding proteins (SREBPs), mitigate hypertension and has hepato- and renal-protective properties; thus, it has a positive effect in the management of obesity and diabetic nephropathy [15,16,17,18,19]. Carnosine also has demonstrated anti-ischemic properties, protecting animals from ischemic heart, kidney, liver and brain injury [20,21,22].

There has been a small number of human trials evaluating the effect of carnosine on cardiometabolic health. In patients with chronic heart failure who were under conventional therapy, carnosine supplementation improved exercise performance and quality of life [23]. Another recently published trial showed that carnosine exerts renal-protective effects by reducing urinary transforming growth factor beta (TGF-β)—a marker of renal injury in patients with diabetic nephropathy [24]. A recent systematic review and meta-analysis (involving 184 participants) showed that triglyceride (TG), total cholesterol (TC), and high-density lipoprotein cholesterol (HDL-C) concentration do not change following carnosine supplementation [25]. However, no study has yet been carried out investigating the effectiveness of carnosine supplementation on blood pressure, arterial stiffness, endothelial function, lipid parameters, and hepatic and renal outcomes in patients with prediabetes and T2DM. Therefore, in the present study, we aimed to evaluate the effect of 14-week carnosine supplementation on important indicators of vascular, renal and hepatic health in those with T2DM and prediabetes.

## 2. Materials and Methods

### 2.1. Study Design and Participants

The present study was a randomized, parallel design, double-blind, placebo-controlled trial. We followed the standard protocol for clinical trials using the Standardised Protocol Interventions: Recommendations for Interventional Trials (SPIRIT) 2013 Statement [26]. Eighty-eight adult patients with prediabetes or T2DM who were untreated, diet-controlled, or on metformin therapy were recruited through community advertising at Monash University and Monash Medical Centre in Melbourne, Australia. Patients were also recruited from the Australian National Diabetes Service Scheme. We screened and recruited participants if they met the following inclusion criteria: 18–70 years old, diagnosed with prediabetes (impaired fasting glycaemia (IFG) which is defined as fasting blood glucose of 6.1–6.9 mmol/L and impaired glucose tolerance (IGT) which is defined as a 2-h blood glucose of 7.8–11.1 mmol/L) or T2DM which was diagnosed based on fasting blood glucose ≥ 7.0 mmol/L and 2-h blood glucose of ≥11.1 mmol/L at screening), hemoglobin A1c (HbA1c) concentration below 8%. If their HbA1c level was below 8%, they were also be recommended not to change their current medication over the course of the trial; stable body weight, with a weight change of no more than 5 kg during the previous six months, and no aim to reduce weight or alter physical activity throughout the study period. Participants were excluded if they were current smokers, had high alcohol use (>2 standard drinks/week for women and >4 standard drinks/week for men), body mass index (BMI) > 40 kg/m^2^, using medications or dietary supplements known to affect cardiometabolic measures, history of blood transfusion within the past three months, renal failure (estimated glomerular filtration rate (GFR) of <30 mL/min), gastrointestinal, hematological, cardiovascular, endocrine or respiratory diseases, psychiatric disorders, central nervous system diseases, active cancer, acute inflammation or infection within the previous five years; and lactating or pregnant women.

### 2.2. Ethics

All included participants provided written informed consent before commencing the trial. The trial was performed in accordance with the principles of the Declaration of Helsinki. The present study received ethical approval from the Human Research Ethics Committee of Monash Health (Ref. No. 16061A) and Monash University (ID number: 7787), Melbourne, Australia. The trial was also registered at clinicaltrials.gov (NCT02917928).

### 2.3. Sample Size Calculation

The sample size determined using GPower (v3.1.9.7) [27] information from a similar cohort of individuals with prediabetes and T2DM who were studied in our laboratory and had a mean change in fasting glucose (mmol/L) of 10 ± 2.0 and HbA1c (%) of 7 ± 0.5. Accordingly, 22 completed participants in each group were required to observe a 20% absolute change in fasting glucose and a 0.5% difference in HbA1c for 80% power. This effect size was clinically significant and comparable to that shown following a 12-week carnosine treatment among subjects with prediabetes [28]. As a result, 50 participants were required to complete the study recruited to account for dropout based on a type I error of 0.05 (two-tailed).

### 2.4. Screening

Following the informed consent process, a medical history was taken, and vital signs and anthropometric measures were assessed by a registered medical practitioner. A urine pregnancy test was used to identify unknowingly pregnant females. Participants then underwent a 2 h OGTT to confirm diabetes or prediabetes, according to the World Health Organization criteria [29]. Screening blood samples were analyzed by Monash Pathology to be analyzed for full blood count, lipid profile, kidney and liver function tests, HbA1c, concentration of calcium, phosphate, and magnesium and C-reactive protein (CRP) as a marker of inflammation.

### 2.5. Intervention and Random Assignment

Following screening participants were randomly assigned to either the carnosine group, in which they were instructed to orally consume four capsules of carnosine daily (CarnoPure, Flamma S.p.A, Italy), or the placebo group, in which they received an equivalent number of identical placebo capsules (two capsules of 500 mg twice daily). The purity of carnosine was greater than 99.5% and it was fully synthetic, odorless, and crystalline. The dose of 2 g per day was based on the previous human trials pilot data on insulin sensitivity and secretion [28]. All participants were asked to make no dietary changes and maintain their usual physical activity. Random assignment was performed using a computerized random-sequence generation program and carried out in blocks of four by gender and metformin treatment to balance between groups. The research statistician created the randomization codes, which were then sent to the clinical trial pharmacy for allocation and dispensing. To ensure double blinding, treatments were dispensed identical, tasteless, non-transparent capsules in clear containers. In order to increase compliance, participants were contacted by phone every four weeks and instructed to return the empty containers at the end of the trial to evaluate compliance to treatment.

### 2.6. Outcome Measures

Outcome measures were obtained at baseline and were repeated after 14-week supplementation. Briefly, participants who were eligible after medical screening were asked to commence their baseline assessment at their second visit. All the procedures that were followed are listed below:

#### 2.6.1. Anthropometric Measurements

A digital scale (Tanita BWB-600, Tanita, Tokyo, Japan) was used to measure body weight (kg), while height (cm) was measured using a stable stadiometer (Seca 206, Seca, Hamburg, Germany), without shoes and lightly clothed. BMI was calculated as weight (kg)/height (m) square.

#### 2.6.2. Blood Pressure

Systolic and diastolic blood pressures, as well as pulse rate were assessed with an automated oscillometric system (Omron BBP-742, Kyoto, Japan), after a 20-min seated rest, as an average of three readings.

#### 2.6.3. Lipid Profile

Lipid profile-related parameters, including plasma TC, TG, high-density lipoprotein cholesterol (HDL-C), and LDL-C were measured using a standard commercial enzymatic assay, on a Beckman Coulter LX20PRO analyzer and SYNCHRON Systems lipid and multi calibrators (Beckman Coulter Diagnostics, Mount Waverley, Victoria, Australia).

#### 2.6.4. Liver Fibroscan

Non-invasive transient elastography (FibroScan^®^, Echo-Sens, Paris, France) was performed to evaluate hepatic fibrosis and steatosis [30]. All FibroScan^®^ measurements were taken by the appropriately trained practitioner. Steatosis was assessed by controlled attenuation parameter (CAP) and liver stiffness was measured in kPa.

#### 2.6.5. Liver Function Tests and Renal Outcomes

Blood samples were taken from participants after at least 10 h fasting with aseptic technique to evaluate liver function tests and renal outcomes. All the blood samples were analyzed by the National Association of Testing Authorities-accredited Monash Health pathology service, which runs an automated core laboratory at Monash Medical Centre Clayton.

#### 2.6.6. Endothelial Function

We used non-invasive peripheral arterial tomography (EndoPAT, Itamar Medical, Caesarea, Israel) to record continuous plethysmographic signals of the finger arterial pulse wave [31]. We put the finger plethysmographic probes on each index finger. A blood pressure cuff on the non-dominant arm was elevated to 200 mmHg for 5 min, then deflated to induce reactive hyperemia. For a further 10 min, post-occlusion alterations, also known as reactive hyperemia PAT (RH-PAT) were measured. Results were adjusted to account for any systemic changes by comparing them to the non-occluded arm (RH-PAT ratio).

#### 2.6.7. Arterial Stiffness and Central Pressure

We used Complior (Alam Medical, Saint Quentin Fallavier, France) to measure aortic (carotid–femoral) pulse wave velocity (aPWV). Pulse transit time was measured and averaged over 10 cycles, with velocity calculated using PWV = D/Δt (m/s), where D (distance) was measured in accordance with the most recent recommendations of the European Working Group on Large Arteries [32]. The Complior was also used to measure the aortic augmentation index and central blood pressure [33,34].

#### 2.6.8. International Physical Activity Questionnaire (IPAQ)

The physical activity level of participants was measured using the short form of IPAQ. The brief IPAQ asks individuals to reflect on the preceding seven days and record the amount of time they spent exercising vigorously (such as aerobics), exercising moderately (such as carrying small loads), walking, and sitting down [35].

#### 2.6.9. Record of Habitual Diet

Dietary intake (food groups, macronutrient, micronutrient, and energy) were assessed through weighed 2 × 3 day food records, comprising three consecutive days (two weekdays and one weekend day). If significant dietary variance was discovered, additional days or non-consecutive days were examined. Foodworks 7 Professional Dietary Software (Xyris Software, Brisbane, Australia) and Australian food composition data were used to analyze food records (NUTTAB 2010).

### 2.7. Statistical Analysis

Analyses were performed per protocol using SPSS Inc., Chicago, USA, version 24, and Graphpad PRISM 8.0 software (Graphpad Software, San Diego, CA, USA). The normality assessment was determined by Shapiro–Wilk tests, scatterplots, and histograms. If normality was violated, continuous variables were transformed by natural logarithm. Descriptive statistics are presented as means ± standard deviations (SD)s, frequencies (percentages), or as median (interquartile ranges (IQRs)) if the distribution was skewed. The effectiveness of the carnosine compared to the placebo on the outcomes (between-group differences) was analyzed through changes in outcome variables and analysis of covariance (ANCOVA) in which baseline values were controlled, and within-group differences were assessed by paired *t*-test. Bonferroni corrections were performed to adjustment for multiple testing. All tests were two-sided, and *p* < 0.05 was considered statistically significant.

## 3. Results

### 3.1. Study Population and Baseline Characteristics

**Figure 1:**
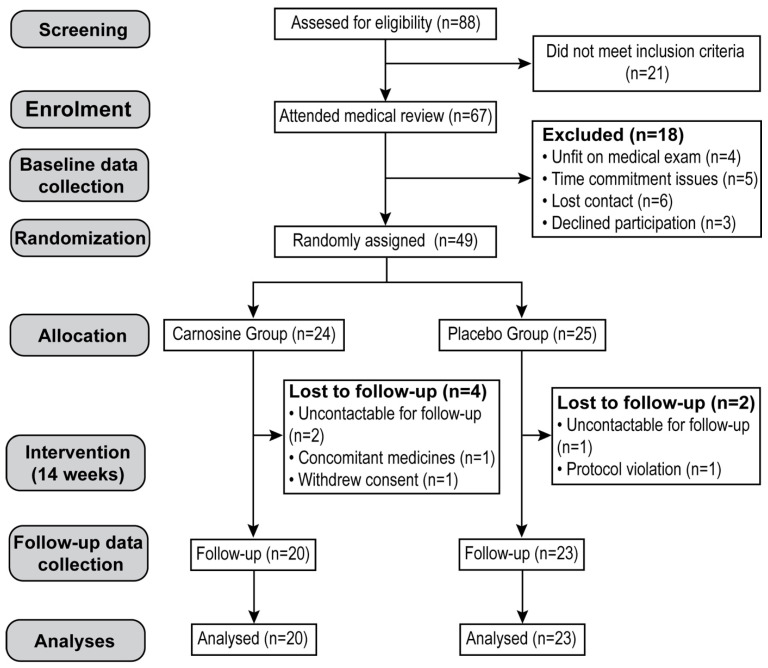
Participant flowchart showing numbers of participants who were recruited, were randomly assigned, dropped out, and were analysed during the trial.

The participant flowchart is presented in Figure 1. Eighty-eight participants were recruited and assessed for eligibility: 21 participants did not meet the inclusion criteria, and the remaining 67 attended medical review. Eighteen participants were subsequently excluded prior to randomization due to time-commitment issues, lost contact, unfitness based on medical exam, or declined participation, leaving 49 who were randomly assigned to receive either carnosine (*n* = 24) or placebo (*n* = 25). By the end of the intervention, six participants dropped out due to a change of medication, protocol violation, were uncontactable for follow-up, or withdrew consent. The study was completed by the remaining 43 participants (20 in the carnosine group and 23 in the placebo group), who were then subjected to per-protocol analysis. Table 1 shows the demographic, anthropometric, and biochemical baseline characteristics of the two groups.

Thirty men and 13 women with a median age (years) of 53 (IQR: 44.2–59.4), mean ± SD of BMI (kg/m^2^) (29.33 ± 4.29), mean ± SD of HOMA-IR (2.60 ± 1.25), mean ± SD of HbA1c (%) (6.6 ± 0.7), mean ± SD of SBP (mmHg) (123.7 ± 12.1), and mean ± SD of DBP (mmHg) (81.3 ± 7.2) completed the study.

### 3.2. Effect of Carnosine Supplementation on Hepatorenal Outcomes

Table 2 shows the efficacy of carnosine supplementation when compared to the placebo on cardiometabolic outcomes before and after the intervention. No significant differences were observed between the carnosine and placebo groups for any of the lipid profile components. Carnosine supplementation improved neither fibroScan^®^-related parameters, including median stiffness (hepatic fibrosis), controlled attenuation parameter (CAP) score (hepatic steatosis), and interquartile range to median stiffness (reliability of hepatic stiffness) nor liver function tests, such as alanine transaminase (ALT), gamma-glutamyl transferase (GGT), and alkaline phosphatase (ALP) as compared with placebo. Similarly, there was no difference between two groups in the changes in renal outcomes which were measured as urea, total bilirubin, sodium, potassium, and bicarbonate levels, and albumin to creatinine ratio (ACR).

### 3.3. Effect of Carnosine Supplementation on Cardiovascular Outcomes

The summarized comparison between carnosine and placebo groups at baseline and after follow-up on cardiovascular outcomes is shown in Table 3. Based on the findings of endo-PAT, in the present study, patients with prediabetes and T2DM showed normal endothelial function (lnRHI > 0.51) before and after the intervention and there was no significant difference between the two groups on endothelial function following the intervention. The change in arterial stiffness, represented by the augmentation index (AIx and AI normalized to the heart rate of 75 bpm) also showed no significant difference between the two groups. Additionally, carnosine did not change short-term heart rate variability (HRV), compared to placebo. Carnosine did not change peripheral and central systolic blood pressure (SBP), diastolic blood pressure (DBP), pulse pressure (PP), or heart rate (HR) as compared to the control group. In addition, no significant differences were seen between the two groups for any of the pulse wave analysis parameters, including carotid-femoral pulse wave velocity (CF PWV) (a marker for arterial stiffness), augmentation index (AI), augmentation pressure (AP), subendocardial viability ratio (SEVR) (index of myocardial oxygen supply and demand), and left ventricular ejection time (LVET).

## 4. Discussion

In this 14-week randomized placebo-controlled trial, we evaluated for the first time, the efficacy of carnosine supplementation in the improvement of endothelial function, arterial stiffness, and indicators of high ASCVD risk in patients with prediabetes and T2DM. Our main outcome adds to the body of emerging evidence demonstrating that 2 g/day carnosine supplementation had no effect on endothelial dysfunction, blood pressure, markers of cardiovascular risk, arterial stiffness, lipid profile, hepatic health indicators, or renal outcomes compared to placebo in patients with prediabetes and T2DM with good glycemic control. As previously reported, carnosine was well-tolerated and we did not observe any significant side effects [28,36,37].

Carnosine is considered a potential therapeutic option that could mitigate the development of cardiovascular events associated with diabetes [38,39,40]. Evidence from experimental studies showed that carnosine administration lowered TG levels [41] and prevented LDL-C oxidation and created stable covalent conjugates with the aldehydes produced during LDL-C oxidation in diabetic apolipoprotein E (^−/−^) mice, inhibiting the progression of atherosclerosis [42]. Early treatment with D-carnosine-octylester (DCO) also protected mice from vascular and renal diseases associated with diabetes through attenuation of progression of macroangiopathy [43]. Another study suggested carnosine as a promising therapeutic agent in humans by its effects on attenuation of renal dysfunction and atherosclerosis in apolipoprotein E-null mice through scavenging reactive carbonyl species (RCS) and 4-hydroxy-2-nonenal (HNE), leading to the decreased carbonylation of proteins and inhibits advanced lipoxidation end products (ALEs) and advanced glycation end products (AGEs) generation and thus reduces inflammation leading to lesion progression [15]. Human intervention studies examining the effectiveness of carnosine supplementation on arterial stiffness are required to examine if these effects are present in humans. We showed that 2 g daily intake of carnosine did not significantly improve the markers related to arterial stiffness in patients with prediabetes and T2DM compared to placebo group. However, the absence of statistical significance for the effect of carnosine intake on arterial stiffness raises the possibility that animal findings may not be translatable to humans. Disparate animal species, different models for inducing disease, and variations in medication dosing and duration are the key problems in the translation of findings from experimental studies to humans. Alternatively, the reason why we saw no differences between the treatment groups is that patients had relatively good glycemic control and well-controlled CVD risk factors.

A recently published review provided evidence regarding the effect of carnosine on endothelial function, but highlighted the need for human studies [44]. Given that elevated levels of oxidative stress and endothelial inflammation and activation (endothelium-leukocyte interaction) are the main contributors to the development of endothelial dysfunction in a variety of cardiovascular diseases, it is reasonable to assume that carnosine intake could have a positive effect on endothelial function [45,46]. In addition, the findings of the trial among young females showed positive effects of the administration of chicken extract, which is a rich source of carnosine, on the reduction of HRV, a parameter that is also related to the presence of endothelial dysfunction and reflects its promising effect on the maintenance of sympatho-vagal balance [47]. There is currently no randomized double-blinded placebo-controlled clinical trial available. In the present study, we did not find any effect of carnosine supplementation on measurements related to endothelial dysfunction in patients with prediabetes and T2DM. This is likely due to the fact that participants receiving carnosine had a low AI value and LnRHI (<0.51) at baseline which reflects the normal arterial elasticity prior to treatment.

Another finding of this study was that the use of carnosine was not associated with the improvement of either central or peripheral blood pressure and pulse pressure and heart rate, while there is a body of emerging evidence on the anti-hypertensive property of carnosine [16,48,49,50]. This is again likely due to normal blood pressure and heart rate at baseline. The effect of carnosine on the histamine/histidine route [51], the NO/cGMP mechanism [52], and the effect on the autonomic nervous system [48,50] have all been proposed as possible explanations for its anti-hypertensive actions. The effects of carnosine on blood pressure may also potentially be due to its long-term effects on atherosclerosis. Our team previously reported that 2 g daily supplementation with carnosine did not significantly change blood pressure [28]. In addition, blood pressure levels did not change following a 13-week chicken meat extract supplementation containing 40% carnosine and anserine in elderly people [53]. However, both studies found that the carnosine-treated group demonstrated significantly decreased heart rate levels. Previous findings showed that lower heart rate is associated with longevity and lower CVD mortality [54]. The mechanism by which carnosine reduced HR was through increasing intracellular Ca^2+^ levels in cardiomyocytes and elevating contractibility [55]. The preservation of vascular tone, platelet aggregation, and endothelial health heavily depends on nitric oxide, which serves as a crucial mediator [44]. It could be hypothesized that since lowered blood pressure was observed in rats after receiving a high dose (33.3 mg/kg) of carnosine intravenously, it is possible that NO generation caused by dietary carnosine may locally dilute blood vessels but does not lower systemic blood pressure [44].

Several animal studies have investigated the effect of carnosine supplementation on lipid parameters and have shown dyslipidemia improvement and reduction of oxidation and glycation of LDL-C following carnosine treatment in both diabetic and non-diabetic rodents [16,56,57]. In addition, L-carnosine supplementation resulted in an increased level of HDL-C in high-fat and high-cholesterol-fed rats [58]. In our pilot study, carnosine supplementation showed beneficial effects on plasma lipidome in overweight and obese adults [59]. Here, we reported no change of lipid profile indicators after carnosine supplementation in subjects with prediabetes and T2DM with good glycemic control. This may be due to the largely normal levels of LDL-C and HDL-C at baseline. Our findings are consistent with the previous results from a systematic review and meta-analysis study [25]. This study included four RCTs showed that carnosine supplementation elicited no effect on TG, TC, and HDL-C. However, we previously showed positive effects on the plasma lipidome [59].

Despite the findings from animal studies that carnosine supplementation resulted in improvement in kidney and liver parameters, we did not find any significant effect of carnosine on fibroScan^®^-related parameters, including median stiffness (hepatic fibrosis), controlled attenuation parameter (CAP) score (hepatic steatosis), and interquartile range to median stiffness (reliability of hepatic stiffness), liver function tests (ALT, GGT, and ALP), and ACR. Results from the study on cirrhotic rats indicated that carnosine supplementation ameliorated hepatic fibrosis and is a promising agent to preserve liver function [60]. In the current study, we did not have any participants with known cirrhosis. Another study showed that histidine and carnosine mitigated high saturated fat-induced hepatic steatosis in mice [18]. Carnosine supplementation was also found to reduce proteinuria and renal damage in diabetic mice [61], a rat model of sepsis [62], and obese Zucker rats [16], and to suppress fibronectin and transforming growth factor β (TGF-β) synthesis in renal cells [63]. Likewise, carnosine administration reduced serum creatinine and urea levels in diabetic rats [64]. Carnosine was also effective in reducing glomerular hypertrophy [65] and podocyte apoptosis and loss [66]. In subjects with diabetic nephropathy, carnosine supplementation could decrease urinary TGF-β levels to serve as a marker of renal injury [24]. In addition, carnosine intake in pediatric patients with type 1 diabetes and nephropathy showed a significant improvement in renal function [67]. This was likely due to a relatively small number of participants and the possibility of not translating experimental findings into human studies.

The present study has several strengths, the main one being that it was the first time that we examined the effectiveness of carnosine supplementation on cardiovascular outcomes using gold-standard endoPAT and Complior devices and other cardiometabolic indicators in individuals with prediabetes and T2DM. Additionally, this study included a population with prediabetes and T2DM. Considering the mentioned strengths, the present study also has some limitations. Firstly, we are aware that the small sample size in our study may have an impact on the analytical power. To prevent bias, all patients were sequentially recruited using strict inclusion/exclusion criteria. Secondly, the moderate dropout rate (12.2%) may have led to a breach of the randomization principles, altering the effect size. To this end, although an intention-to-treat analysis would be favorable, we conducted per-protocol analysis which is preferred in trials with small sample size [68]. Because of these factors, results should be evaluated carefully. Thirdly, most of the patients at baseline had normal levels of cardiovascular and cardiometabolic outcomes, so our findings may not apply to other populations because we only included people with prediabetes and T2DM.

## 5. Conclusions

We showed for the first time that carnosine supplementation has no beneficial effects on cardiovascular parameters, including arterial stiffness and endothelial dysfunction, or other cardiometabolic disease indicators, such as blood pressure, heart rate, kidney and liver function, and lipid parameters in adults with normal levels of cardiovascular and cardiometabolic parameters. Whether carnosine supplementation can reduce ASCVD risk or improve metabolic outcomes in patients with prediabetes and T2DM with more elevated cardiovascular risk factors needs to be confirmed.

## Figures and Tables

**Table 1:** Participant demographics and baseline characteristics.

Characteristic	Carnosine Group (*n* = 20)	Placebo Group (*n* = 23)
Age, year	53.7 (48.5–59.4) ^a^	52 (42.3–59.3)
Men, *n* (%)	14 (70)	16 (69.56)
Caucasian	11 (55)	11 (47.82)
South and Central Asian	6 (30)	6 (26.08)
Southeast and Northeast Asian	3 (15)	5 (21.73)
Other ^b^	-	1 (4.34)
Total energy, kj	7935.80 ± 1303.64	8206.88 ± 1319.38
Physical activity ^c^, IPAQ-METS score ^d^	2400 (798–4878)	1332 (390–2736)
Weight, kg	86.92 ± 21.22	82.02 ± 13.61
BMI, kg/m^2^	30.54 ± 4.74	28.29 ± 3.66
HOMA-IR	2.88 ± 1.39	2.35 ± 1.08
HbA1c, %	6.48 ± 0.63	6.65 ± 0.83
SBP, mmHg	124.94 ± 12.97	122.62 ± 11.46
DBP, mmHg	81.94 ± 8.59	80.71 ± 5.81
Family history of diabetes ^e^, *n* (%)	5 (25)	4 (17.39)
Patients with T2DM, *n*	11 (55)	17 (73.91)
Dyslipidaemia, *n*	2 (10)	4 (17.39)
Nephropathy, *n*	2 (10)	2 (8.69)
Oral glucose-lowering agents, *n*	7 (35)	7 (30.43)
Diet therapy, *n*	5 (25)	10 (43.47)
No treatment, *n*	8 (40)	6 (26.08)

^a^ Median; IQR in in parentheses (all such values for nonnormally distributed variables). Nonnormally distributed variables were log transformed to the base 10 before analysis. ^b^ Includes African, Middle Eastern, South American, and Polynesian ethnicities. ^c^ Calculated from self-reported questionnaires and food records. ^d^ IPAQ-METS, international physical activity questionnaire–multiples of the resting metabolic rate. ^e^ Includes only first-degree relative with diabetes. Abbreviations. IPAQ-METS: International physical activity questionnaire-metabolic equivalent, BMI: Body mass index, HOMA-IR: Homeostatic model assessment for insulin resistance, HbA1c: Hemoglobin A1c, SBP: Systolic blood pressure, DBP: Diastolic blood pressure, and T2DM: Type 2 diabetes mellitus.

**Table 2:** Comparison of cardiometabolic outcomes before and after supplementation in both groups.

Outcome Variable		Carnosine Group (*n* = 20)				Placebo Group (*n* = 23)				
		Baseline	Follow-Up	*p* ^a^	Change	Baseline	Follow-Up	*p* ^b^	Change	*p* ^c^
Lipid profile	TG (mmol/L)	1.66 ± 0.88 ^d^	1.58 ± 0.73	>0.99	−0.01	1.72 ± 0.67	1.86 ± 0.75	>0.99	0.02	0.231
	TC (mmol/L)	5.36 ± 0.89	5.29 ± 0.71	>0.99	−0.003	5.30 ± 0.97	5.14 ± 0.85	>0.99	−0.01	0.428
	LDL-C (mmol/L)	3.36 ± 0.6	3.42 ± 0.57	>0.99	0.05	3.19 ± 0.6	3.04 ± 0.55	>0.99	−0.14	0.060
	HDL-C (mmol/L)	1.13 ± 0.26	1.1 ± 0.23	>0.99	−0.03	1.19 ± 0.26	1.15 ± 0.26	>0.99	−0.03	0.810
	LDL-C/HDL-C	3.1 ± 0.85	3.15 ± 0.79	>0.99	0.11	2.81 ± 0.68	2.68 ± 0.75	>0.99	−0.13	0.100
Hepatic outcomes	ALT (U/L)	38.32 ± 18.64	36 ± 15.50	>0.99	−2.32	38.19 ± 16.34	34.33 ± 15.55	0.39	−3.86	0.321
	GGT (U/L)	34.47 ± 13.35	34.53 ± 12.90	>0.99	0.05	34.33 ± 12.57	32.57 ± 12.13	>0.99	−1.76	0.476
	ALP (U/L)	82.63 ± 18.23	79.42 ± 16.13	>0.99	−3.21	79.57 ± 22.06	75.48 ± 18.35	>0.99	−4.10	0.569
	IQR	33.17 ± 9.57	27.17 ± 11.28	>0.99	−6	34.53 ± 14.01	30.53 ± 11.39	>0.99	−4	0.470
	Median stiffness (KPa)	5.66 ± 2.00	5.72 ± 1.59	>0.99	0.01	5.13 ± 1.64	5.51 ± 1.24	>0.99	0.04	0.826
	IQR to Median stiffness	0.13 ± 0.06	0.15 ± 0.07	>0.99	0.02	0.13 ± 0.06	0.13 ± 0.05	>0.99	0.004	0.390
	CAP (dB/m)	290.6 ± 72.21	301 ± 61.86	>0.99	10.4	288.56 ± 61.04	293.78 ± 64.68	>0.99	5.22	0.655
Renal outcomes	Sodium (mmol/L)	139 (137–140) ^e^	138 (137–139)	>0.99	−0.21	138 (137–140)	138.5 (137–140)	>0.99	0.09	0.521
	Potassium (mmol/L)	4.23 ± 0.26	4.16 ± 0.22	>0.99	−0.06	4.20 ± 0.25	4.19 ± 0.20	>0.99	−0.009	0.555
	Bicarbonate (mmol/L)	25.63 ± 2.21	25.63 ± 2.40	>0.99	0	25.91 ± 1.74	25.68 ± 2.12	>0.99	−0.23	0.763
	Urea (mmol/L)	5.24 ± 0.87	5.43 ± 1.14	>0.99	0.18	5.18 ± 1.21	5.68 ± 1.54	0.91	0.5	0.385
	Total bilirubin (µmol/L)	11.63 ± 3.71	12.32 ± 3.93	>0.99	0.68	12.67 ± 3.90	12.14 ± 4.54	>0.99	−0.52	0.200
	Albumin (g/L)	40.37 ± 3.27	39 ± 2.90	0.13	−1.37	41.29 ± 2.75	40.24 ± 1.94	>0.99	−1.05	0.198
	Creatinine (µmol/L)	64.21 ± 8.86	66.63 ± 8.00	0.37	2.42	71.86 ± 15.99	73.27 ± 16.68	>0.99	1.41	0.765
	ACR	9.60 ± 1.37	8.91 ± 1.17	0.007	−0.69	8.99 ± 2.12	8.58 ± 2.06	0.32	−0.41	0.541

^a^ Determined with the use of paired Student’s *t* tests for differences between baseline and follow-up and were adjusted for multiple testing with the use of Bonferroni correction in the carnosine group. ^b^ Determined with the use of paired Student’s *t* tests for differences between baseline and follow-up were adjusted for multiple testing with the use of Bonferroni correction in the placebo group. ^c^ Determined with the use of a multiple linear regression analysis (ANCOVA) for differences between carnosine and placebo groups after adjustment for baseline values. ^d^ Mean ± SD (all such values). ^e^ Median; IQR in in parentheses (all such values for nonnormally distributed variables). Nonnormally distributed variables were log transformed to the base 10 before analysis. Abbreviations. ALT: Alanine transaminase, GGT: Gamma-glutamyl transferase, ALP: Alkaline phosphatase, IQR: Interquartile range, CAP: Controlled attenuation parameter, and ACR: Albumin to creatinine ratio.

**Table 3:** Comparison of cardiovascular outcomes before and after supplementation in both groups.

Outcome Variable		Carnosine Group (*n* = 20)				Placebo Group (*n* = 23)				
		Baseline	Follow-Up	*p* ^a^	Change	Baseline	Follow-Up	*p* ^b^	Change	*p* ^c^
EndoPAT	SBP (mmHg)	122.76 ± 14.77 ^d^	121.24 ± 13.17	>0.99	−1.53	122.72 ± 12.48	122.94 ± 16.41	>0.99	0.22	0.667
	DBP (mmHg)	80.65 ± 8.25	79.59 ± 7.59	>0.99	−1.47	82.11 ± 7.07	82.50 ± 9.40	>0.99	0.67	0.421
	BMI (kg/m^2^)	29.73 ± 4.28	29.68 ± 4.31	>0.99	−0.09	27.81 ± 2.88	27.73 ± 3.08	>0.99	−0.07	0.981
	LnRHI	1 ± 0.2	0.75 ± 0.22	>0.99	−0.24	0.96 ± 0.68	0.68 ± 0.28	>0.99	−0.27	0.437
	HR (bpm)	65.53 ± 9.50	67.12 ± 7.83	>0.99	1.76	69.61 ± 11.34	68.94 ± 6.83	>0.99	0.39	0.923
	AI%	2.24 ± 8.12	5.53 ± 13.24	>0.99	3.53	7.61 ± 10.01	4.89 ± 18.64	>0.99	−2.39	0.321
	AI@75bpm (%)	0.24 ± 12.26	2.24 ± 12.60	>0.99	2.24	7.35 ± 14.12	7.18 ± 12.25	>0.99	2.41	0.579
	HRV (ms)	29.85 ± 12.01	30.27 ± 10.92	>0.99	0.41	33.57 ± 6.31	36.57 ± 13.19	>0.99	2.99	0.420
	LF (m/s^2^)	224.05 ± 64.71	190.71 ± 74.23	>0.99	−33.33	185.10 ± 77.38	135.38 ± 55.18	>0.99	−49.71	0.132
	HF (m/s^2^)	88.70 ± 50.17	126.06 ± 59.64	>0.99	37.36	123.21 ± 36.25	125.94 ± 74.14	>0.99	2.72	0.570
	LF/HF	3.05 ± 1.34	1.82 ± 0.94	0.08	−1.22	1.78 ± 1.51	1.27 ± 0.82	>0.99	−0.42	0.562
Complior	Peripheral BP									
	SBP (mmHg)	120.44 ± 15.37	124.07 ± 10.37	>0.99	3.63	120.03 ± 11.87	120.9 ± 12.16	>0.99	0.86	0.334
	DBP (mmHg)	78.6 ± 8.6	79.03 ± 5.77	>0.99	0.37	80.72 ± 6.56	79.04 ± 8.51	>0.99	−1.67	0.724
	MAP (mmHg)	93.05 ± 9.88	94.24 ± 5.71	>0.99	1.18	93.57 ± 7.25	92.7 ± 8.39	>0.99	−0.85	0.451
	PP (mmHg)	42.27 ± 10.38	45.23 ± 11.26	>0.99	2.96	39.25 ± 10.39	41.80 ± 8.50	>0.99	2.55	0.573
	HR (bpm)	63.17 ± 8.82	67.41 ± 9.84	>0.99	4.24	70 ± 10.77	70.67 ± 8.94	>0.99	0.67	0.985
	Central BP									
	cSBP (mmHg)	114.74 ± 17.74	116.08 ± 10.89	>0.99	1.34	115.3 ± 14.56	110.67 ± 13.50	>0.99	−4.65	0.095
	cDBP (mmHg)	78.81 ± 8.91	79.23 ± 6.09	>0.99	0.42	81 ± 7.06	80.1 ± 8.30	>0.99	−0.9	1.00
	cPP (mmHg)	36.42 ± 10.7	38.37 ± 10.23	>0.99	1.94	34.45 ± 12.51	32.01 ± 9.53	>0.99	−2.43	0.123
	PP amplification (mmHg)	1.19 ± 0.23	1.22 ± 0.27	>0.99	0.02	1.18 ± 0.21	1.35 ± 0.23	>0.99	0.17	0.127
	Pulse wave analysis									
	CF PWV (m/s)	7.4 (6.2–8.5) ^e^	7.6 (2.4–8.5)	>0.99	−1.19	7.1 (5.7–7.8)	7.1 (6.5–8.4)	>0.99	0.23	0.123
	Alx (%)	13.9 (−13.8–18.9)	8.2 (−17.8–15.7)	>0.99	−6.87	9.2 (−26.9–25.3)	13.1 (−7.6–25)	>0.99	9.07	0.339
	AP (mmHg)	9.6 (4.5–12.3)	7.6 (4.8–14)	>0.99	−0.46	7.5 (4.2–16.5)	8 (3.2–15.7)	>0.99	−1.8	0.585
	SEVR (%)	105.22 ± 46.60	99.05 ± 42.24	>0.99	−6.17	102.28 ± 50.23	109.62 ± 47.36	>0.99	7.33	0.482
	LVET (ms)	415.51 ± 80.20	430.90 ± 121.32	>0.99	15.38	406.62 ± 135.64	386.25 ± 86.80	>0.99	−20.37	0.227
	Max dp/dt (mmHg/s)	720 (499.9–940)	896.6 (643.3–1021.6)	>0.99	110.42	584.1 (456.6–793.7)	661.6 (343.3–822.5)	>0.99	−76.85	0.055

^a^ Determined with the use of paired Student’s *t* tests for differences between baseline and follow-up and were adjusted for multiple testing with the use of Bonferroni correction in the carnosine group. ^b^ Determined with the use of paired Student’s *t* tests for differences between baseline and follow-up were adjusted for multiple testing with the use of Bonferroni correction in the placebo group. ^c^ Determined with the use of a multiple linear regression analysis (ANCOVA) for differences between carnosine and placebo groups after adjustment for baseline values. ^d^ Mean ± SD (all such values). ^e^ Median; IQR in in parentheses (all such values for nonnormally distributed variables). Nonnormally distributed variables were log transformed to the base 10 before. Abbreviations. SBP: Systolic blood pressure, DBP: Diastolic blood pressure, BMI: Body mass index, LnRHI: Logarithm of reactive hyperemia, HR: Heart rate, AI%: Augmentation index, HRV: Heart rate variability, LF: Low frequency, HF: High frequency, MAP: Mean arterial pressure, PP: Pulse pressure, CF PWV: Arterial stiffness (carotid femoral pulse wave velocity, AP: Augmentation pressure, SEVR: Subendocardial viability ratio, LVET: Left ventricular ejection time (LVET), and Max dp/dt: Maximal rate of rise of left ventricular pressure.

## Data Availability

The datasets used and/or analyzed during the current study are available from the corresponding author upon reasonable request.
